# Prediction of the *P. falciparum* Target Space Relevant to Malaria Drug Discovery

**DOI:** 10.1371/journal.pcbi.1003257

**Published:** 2013-10-17

**Authors:** Andreas Spitzmüller, Jordi Mestres

**Affiliations:** Chemotargets SL and Systems Pharmacology, Research Programme on Biomedical Informatics (GRIB), IMIM Hospital del Mar Research Institute and Universitat Pompeu Fabra, Parc de Recerca Biomèdica, Barcelona, Catalonia, Spain; University of Heidelberg, Germany

## Abstract

Malaria is still one of the most devastating infectious diseases, affecting hundreds of millions of patients worldwide. Even though there are several established drugs in clinical use for malaria treatment, there is an urgent need for new drugs acting through novel mechanisms of action due to the rapid development of resistance. Resistance emerges when the parasite manages to mutate the sequence of the drug targets to the extent that the protein can still perform its function in the parasite but can no longer be inhibited by the drug, which then becomes almost ineffective. The design of a new generation of malaria drugs targeting multiple essential proteins would make it more difficult for the parasite to develop full resistance without lethally disrupting some of its vital functions. The challenge is then to identify which set of *Plasmodium falciparum* proteins, among the millions of possible combinations, can be targeted at the same time by a given chemotype. To do that, we predicted first the targets of the close to 20,000 antimalarial hits identified recently in three independent phenotypic screening campaigns. All targets predicted were then projected onto the genome of *P. falciparum* using orthologous relationships. A total of 226 *P. falciparum* proteins were predicted to be hit by at least one compound, of which 39 were found to be significantly enriched by the presence and degree of affinity of phenotypically active compounds. The analysis of the chemically compatible target combinations containing at least one of those 39 targets led to the identification of a priority set of 64 multi-target profiles that can set the ground for a new generation of more robust malaria drugs.

## Author Summary

There is an urgent need for new antimalarials acting through novel mechanisms of action that can overcome the increasing incidence of resistance observed for currently used drugs. In this respect, drug polypharmacology is emerging as an attractive strategy to reduce the chances of the parasite evolving drug resistance. Although there were close to 20,000 antimalarial hits recently identified from three independent phenotypic screenings, the molecular targets through which most of those active compounds exert their antimalarial action are unfortunately unknown at present. To address this issue, a computational approach was first used to predict their protein targets. Statistical analyses were applied to detect any enrichment of phenotypically active compounds in *P. falciparum* targets, leading to a final list of 39 putative high-priority targets for malaria drug discovery. The presence of at least one high-priority target in the target profile predicted for the antimalarial hits was then used as a constraint to identify a priority set of 64 multi-target profiles. Multi-target strategies based on such profiles can set the basis for designing a next generation of more robust malaria drugs.

## Introduction

**Figure 1:**
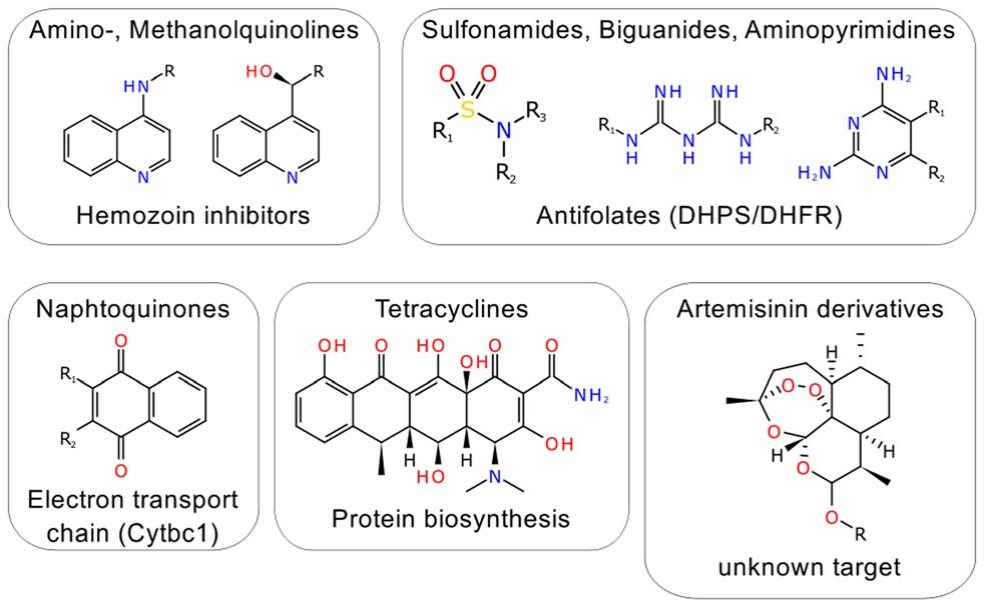
Clinically used antimalarial chemotypes and their corresponding MoA.

Malaria has become one of the most devastating infectious diseases in the world. Just in 2010, it affected about 219 million patients resulting in 660,000 deaths [1]. The disease is caused by several different parasites of the genus *Plasmodium*, with *Plasmodium falciparum* being the most lethal one. Today, there are mainly eight different chemotypes in clinical use for malaria treatment that act through essentially five different mechanisms of action (Figure 1) [2]. However, drug resistant strains of the parasite are currently known for all these mechanisms [3]. Even more worrying is the fact that increasing emergence of multi-drug resistance has been observed in the last decade [4]. Therefore, there is an urgent need for new drugs acting through novel modes of action (MoA) for malaria treatment.

In the past few years, there have been strong demands to generate and integrate molecular, functional and pharmacological data into a common malaria-related chemogenomic knowledge space [5]. Several initiatives have contributed greatly to promote such community efforts to facilitate the discovery of a next generation of malaria drugs. Cell-based high throughput screening campaigns were conducted independently by groups both from academia (St. Jude Children's Research Hospital) and industry (GSK, Novartis) [6]. They all used phenotypic assays against intraerythrocytic *P. falciparum* stages. The St. Jude group screened a set of almost 310,000 commercially available compounds, resulting in 1,134 validated hits [7]. At GSK, the screening was performed against an internal collection of about two million compounds, yielding 13,519 confirmed hits known as the Tres Cantos Antimalarial Set (TCAMS) [8]. At Novartis, a screen of about 800,000 non-proprietary compounds identified 5,697 confirmed hits [6], [9]. In addition to the identified phenotypically active hits, the St. Jude group disclosed the list of 308,324 compounds found to be inactive in the phenotypic assay. In total about 3.1 million compounds, both commercial and proprietary, were tested, leading to the identification of close to 20,000 phenotypically active compounds. Most importantly, the results were then made publicly available at the ChEMBL – Neglected Tropical Disease archive (ChEMBL-NTD), an invaluable resource for current malaria research [10]. This can certainly be considered an important milestone in malaria research, as for the first time a large amount of molecules with phenotypic data are available to the academic community to facilitate global antimalarial drug discovery efforts.

The advantages of using cell-based over target-directed screenings in malaria drug discovery were recently highlighted by Chatterjee and Yeung [11]. In particular, the large chemical diversity among the identified compounds and their immanent favourable properties, such as good solubility and permeability, are widely recognized. Compounds found to be active in a whole-cell assay demonstrate their intrinsic ability to penetrate the parasite and to inhibit essential targets or pathways *in vivo*. In addition, compared to the inherent mechanistic limitations of hits obtained from target-directed campaigns, phenotypically active compounds offer the possibility to exert their action through multiple protein targets, the synergistic combination of which is of *a priori* unknown relevance to malaria.

In contrast, one of the major disadvantages of whole-cell assays over target-based assays is the unknown exact MoA of the identified hits. Without this knowledge, hit progression becomes extremely challenging. To address this issue, means to experimentally determine the protein targets likely to be hit by an active molecule are applied, but they are generally rather cost- and time-consuming and can therefore be conducted only for a limited set of carefully selected targets [12], [13]. Therefore, any efficient strategy that could assist in predicting and prioritizing the target space covered by phenotypically active compounds would represent a significant step forward in the applicability of cell-based assays in malaria drug discovery.

A first attempt in this direction was reported by Plouffe *et al.* [14] Although molecular targets were not explicitly identified, they described a method based on MeSH terms to estimate the MoA of antimalarials found in a phenotypic screen using a guilt-by-association strategy. For each compound, an activity profile was first compiled using data obtained from 131 historical, mostly cell-based, assays. Then, all hits were assigned to a scaffold family and, whenever possible, a MoA annotation was added from the MeSH database. Finally, these two pieces of information were combined to obtain clusters enriched with hits having similar activity profiles. Overall, 31 MeSH groups were identified containing compounds with significantly more similar profiles than what was expected by chance.

Hypotheses for the MoA of the TCAMS screening hits were also generated by Gamo *et al.* after an analysis of GSK's historical activity data in assays against human and microbial targets [8]. To identify targets preferentially inhibited by TCAMS compounds compared to all historically screened compounds, the ratio of known inhibitors among the hits over the total number of inhibitors among all compounds was calculated for each target. Only targets that were at least twofold enriched by antimalarial hits were considered relevant. Searching for homologues of these known targets in the *P. falciparum* genome yielded a set of 51 potential antimalarial targets assigned to a total of 555 hits. The most prominent protein families targeted by TCAMS were kinases and proteases with thirty and ten members, respectively. More recently, Jensen *et al.* described a combination of similarity-based virtual screening and protein homology analysis to predict the molecular targets of TCAMS hits [15]. They were able to assign a total of 293 *P. falciparum* proteins to a set of 4,495 hits. The identified targets were then further prioritized according to criteria like essentiality, lack of human homologues, or favourable polypharmacology profiles.

The aim of the present study is to predict and prioritize the set of *P. falciparum* targets most likely to be hit by the thousands of phenotypically active compounds deposited in the ChEMBL-NTD repository by the St. Jude, GSK and Novartis screening campaigns. Identification of *P. falciparum* targets was performed by predicting first the targets for all phenotypically active and inactive compounds using a ligand-based *in silico* approach [16] and projecting them by orthology onto the *P. falciparum* genome. Prioritization of *P. falciparum* targets was ultimately achieved by applying statistical tests to identify targets enriched with phenotypically active compounds among all their predicted ligands or biased with higher predicted affinity values for their assigned phenotypically active compounds compared to the inactive ones. Combining these two tests, a final list of 39 high priority *P. falciparum* targets was obtained and its potential impact for future multi-target malaria drug discovery will be discussed.

## Results

### Prediction of the *P. falciparum* target space

In recent years, thousands of phenotypically active antimalarials have been identified from three independent sources and made publicly available in the ChEMBL-NTD archive. However, due to the cell-based nature of the underlying screening assays, the exact molecular MoA leading to antimalarial activity of these hits is unknown at present. In an attempt to address this current deficiency, *in silico* target profiling was applied to 18,955 unique active and 303,961 unique inactive compounds identified in cell-based screenings (see Materials and Methods for further details). Predicted interactions to at least one protein target were returned for 48.6% and 66.0% of the active and inactive compounds, respectively. This means that up to 51.4% and 34.0% of the active and inactive compounds, respectively, were found outside the applicability domain of the ligand-based target models used [16]. This observed difference in chemical coverage between active and inactive compounds reflects the fact that all the inactive compounds originate from the St. Jude library, composed entirely of commercially available molecules. In contrast, many of the phenotypically active compounds, strongly dominated by GSK's TCAMS, come from proprietary libraries within pharmaceutical companies and thus are less well covered by molecules present in chemogenomic databases from which the applied models are constructed.

In addition, since the main aim of this study is to identify the likely primary targets for those compounds, any protein-ligand interaction with a predicted affinity value above 10 µM (that is, a value of −log(affinity) below 5) was not considered relevant at this stage. This reduced the number of active and inactive compounds with at least one relevant target interaction predicted by 7.0% and 14.3%, respectively. In the end, a total of 8,556 and 171,830 phenotypically active and inactive compounds, respectively, remained and defined the basis on which all subsequent analyses were performed.

Biologically relevant interactions to 1,288 and 1,543 unique proteins were predicted for the phenotypically active and inactive molecules, respectively. However, since most of the publically available interaction data is measured on human proteins, the vast majority of these predicted proteins are human targets. Accordingly, an orthologous mapping from the predicted targets to the *P. falciparum* genome was performed using OrthoMCL-DB version 5 [17]. At present, this database clusters the genomes of 150 species, including *P. falciparum*, into groups of orthologous genes. Additionally, a web service is offered to assign custom gene products to those groups. Using this service, we were able to map 207 out of the 1,288 targets predicted for the active compounds to 147 *P. falciparum* proteins. An additional set of 79 *P. falciparum* proteins was obtained from the inactive compounds. In total, 226 *P. falciparum* targets could be mapped by orthology to any of the originally predicted targets, a target space that can be considered an approximation to the druggable genome of *P. falciparum*.

In this respect, it ought to be stressed here that this approximation to the druggable *P. falciparum* genome is largely conservative due to the fact that the applied ligand-based approach can only produce a prediction if there are already ligands for this or any orthologous target known in publicly available databases. Indeed, from the 5,524 *P. falciparum* genes listed in OrthoMCL only 574 (10.4%) could be mapped to a target model for which at least one ligand is reported in public repositories. Since we cannot state anything at this stage about the remaining 89.6% of the *P. falciparum* genome, its potential druggable part might well be larger. The complete list of 226 *P. falciparum* targets and the corresponding predicted compounds is available as Supporting Information (Table S1).

**Figure 2:**
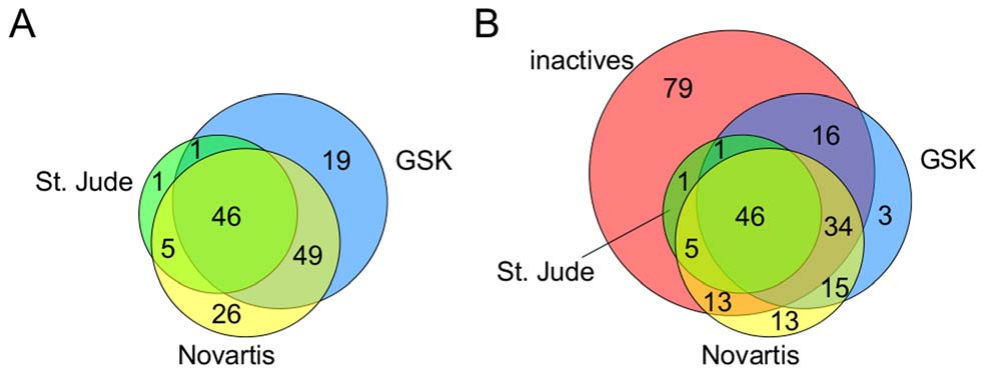
Contributions of the individual data sets to the predicted target space. (A) Target space of active antimalarials only; (B) target space including both active and inactive compounds.

While the predicted druggable *P. falciparum* genome contains 226 targets, not all of them are addressed by a compound from the set of phenotypically active molecules. Therefore, we delimited the relevant target space with respect to malaria drug discovery to those 147 targets predicted for at least one phenotypically active compound. Figure 2A shows the contributions of the individual data sources to the predicted target space. Remarkably, 46 out of the 147 identified targets (31.3%) are coming from active compounds identified in all three screened libraries, an additional set of 55 targets (37.4%) are coming from at least two of them. Thus, even though the phenotypic screenings were performed on three independent libraries, they seem to have a substantial degree of overlap in the target space addressed by their compounds. This result agrees well with the observation made recently by Guiguemde *et al.* that, at the scaffold level, the three chemical libraries are highly similar [6].

The mere fact that a protein is predicted to interact with a compound found active in a phenotypic malaria screening is not sufficient to qualify it as a relevant *P. falciparum* target. Thus, we aimed to reduce the set of 147 *P. falciparum* proteins predicted to interact with at least one phenotypically active compound to a limited number of most highly relevant targets for malaria. To this end, we made for the first time use of the wealth of information inherent in the available negative data, the set of compounds found to be inactive against the parasite cells in the St. Jude screening campaign. The overlap between the target spaces of the phenotypically active and inactive compounds is shown in Figure 2B. As can be observed, most of the predicted proteins are addressed by both active and inactive compounds, while there are only 31 proteins that seem to be interacting exclusively with phenotypically active compounds identified from the Novartis and/or GSK screenings. Thus, in order to identify those proteins most likely to be relevant malaria targets we looked for, on the one hand, targets that are preferentially addressed by phenotypically active compounds and, on the other hand, targets for which the assigned active compounds tend to be predicted with higher affinity values compared to the assigned inactive ones. or both The basic assumption is that targets conforming to either of these criteria are much more likely to be relevant drug targets for malaria.

### Target space enriched with phenotypically active compounds

**Figure 3:**
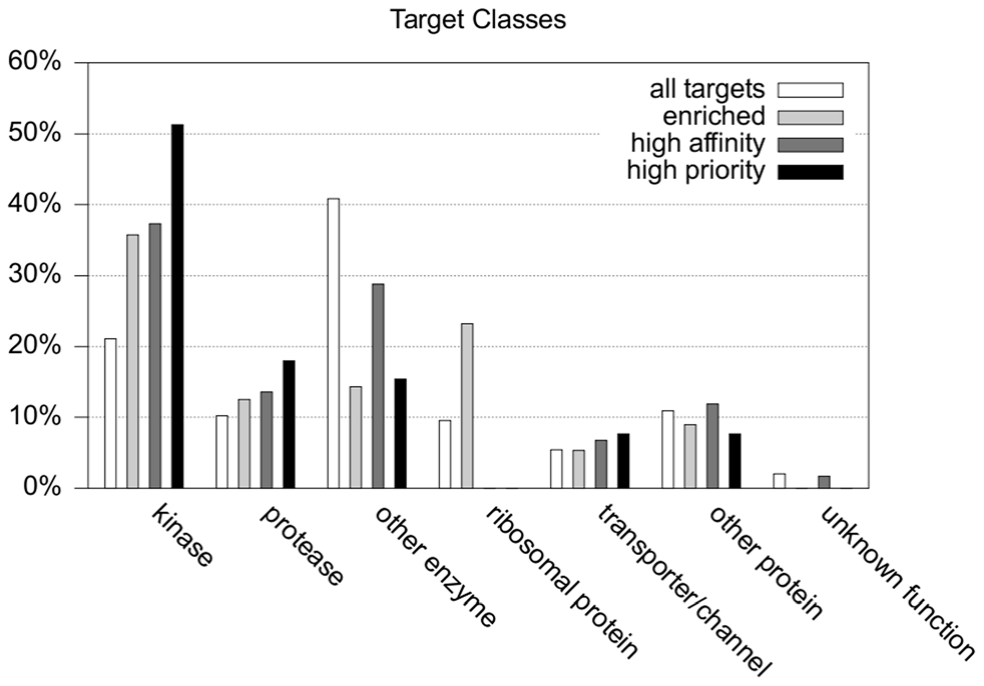
Protein classes of predicted targets. White bars represent the predicted relevant target space, whereas the different prioritized subsets are depicted by shaded bars.

An analysis of the distribution of the 147 *P. falciparum* targets among different protein families (Figure 3, white bars) reveals that the vast majority (72%) are enzymes. Among them, kinases and proteases dominate with 21% and 10%, respectively. On this basis, a statistical test was applied to decide if, for any given target, the predicted compounds from the active set were significantly enriched over the inactive ones. Assuming that compounds from both sets were assigned uniformly to a given target, on average about 6% of phenotypically active compounds could be expected among all predictions, which corresponds to the amount of actives among all screened molecules. This uniform distribution defines the null-hypothesis for this test. Then, for each target with a total of *n* predictions and *k* actives among them, the probability *p*(*k*,*n*) of getting at least *k* actives among *n* predictions was calculated using a permutation test based on this null-hypothesis. Each target for which at least one orthologous model was enriched with phenotypically active compounds at a significance level of 99.9% (*p*<10^−3^) was then selected as a significantly enriched target.

A total of 56 targets enriched with phenotypically active compounds were identified. In the remainder of this work, they will be referred to as the set of “enriched targets”. Among them, 36% were kinases and 23% ribosomal proteins (Figure 3, light grey bars). These numbers represent a considerable increase in the relative importance of these families among the enriched targets compared to the original set of 147 *P. falciparum* targets (Figure 3, white bars). In contrast, the number of proteins in the “other enzymes” category was severely reduced from 41% to 14% suggesting activity on those enzymes alone might not be sufficient to observe a growth limiting effect on the parasite.

### Target space biased with high affinity predictions for phenotypically active compounds

In the previous analysis, targets were prioritized based on enrichment of phenotypically active compounds among all compounds predicted to interact with them without considering the actual predicted affinity values. To further exploit this information statistical tests were applied that allow for the identification of targets that have a significant bias towards higher predicted affinity values for phenotypically active compounds compared to the inactive ones.

Accordingly, all predictions for a target were divided into two sets of phenotypically active and inactive compounds. Then, a one-sided Wilcoxon rank-sum test was applied with the null-hypothesis that the predicted affinity values were accumulating around the same location for both samples and the alternative hypothesis that affinity predictions for the active compounds were shifted towards higher values. Again a significance level of 99.9% (*p*<10^−3^) was used to select those targets for which the null-hypothesis could be rejected.

Under these criteria, a second set of 59 targets was identified as having a significant bias towards higher affinity predictions for phenotypically active compounds compared to inactive compounds (Figure 3, dark grey bars). In the remainder of this work, they will be referred to as the set of “high affinity targets”. Following the trend observed for the enriched targets, the number of kinases in this set was again relatively high (37%), followed by those proteins clustered in the “other enzymes” category (29%). In contrast, ribosomal proteins disappeared completely in this set due to the fact that only qualitative information on protein-ligand interaction data (active or inactive) was available in chemogenomic databases for these proteins and thus, the rank-sum test could not be applied.

### High priority antimalarial target space

The final list of 39 *P. falciparum* targets that, based on currently available data, may be considered of highest priority for malaria drug discovery consists of the intersection between the two sets of enriched and high affinity targets. The relative importance of the different protein families within this final list of “high priority targets” (Figure 3, black bars) is strongly dominated by kinases (51%) followed, at a significant distance, by proteases (18%). The complete list of 39 high priority targets is provided in Table 1.

**Table 1:** List of predicted 39 high priority malaria targets and their TDR ranking.

ID^a^	Protein	TDR Ranking
**Kinases:**		
PFL1490w	atypical protein kinase, RIO family, putative	1536
*PFB0815w*	calcium dependent protein kinase 1 (CDPK1)	409
*PFF0520w*	calcium-dependent protein kinase (CDPK2)	180
*PFC0420w*	calcium dependent protein kinase 3 (CDPK3)	143
PF07_0072	calcium dependent protein kinase 4 (CDPK4)	180
*PF13_0211*	calcium dependent protein kinase 5 (CDPK5)	180
PFI1685w *	cAMP-dependent protein kinase catalytic subunit (PKAc)	27
PFL1110c	cAMP-dependent protein kinase regulatory subunit (PKAr)	857
*PF11_0377*	casein kinase 1 (CK1)	16
PF14_0346 *	cGMP-dependent protein kinase (PKG)	69
PF14_0294	mitogen-activated protein kinase 1 (MAP1)	637
PFA0515w	phosphatidylinositol-4-phosphate 5-kinase (PIP5K)	637
MAL13P1.279 *	protein kinase 5 (PK5)	3
PFB0150c	protein kinase, putative	914
PFL2250c *	RAC-beta serine/threonine protein kinase (PKB)	27
*PFC0385c*	serine/threonine protein kinase, putative (ARK2)	341
*PF14_0516*	serine/threonine protein kinase, putative (KIN)	710
*PF11_0488*	serine/threonine protein kinase, putative	1536
PFL2280w	serine/threonine protein kinase, putative	379
*PFC0105w*	serine/threonine protein kinase (SRPK1)	637
**Proteases:**		
PF11_0165 *	cysteine proteinase falcipain 2a	10
PF11_0161 *	cysteine proteinase falcipain 2b (FP2B)	50
PF11_0162 *	cysteine proteinase falcipain 3	50
PF14_0076 *	plasmepsin I (PMI)	39
PF14_0077 *	plasmepsin II	39
PF14_0075 *	plasmepsin IV (PM4)	39
PFC0495w *	plasmepsin VI	86
**Other Enzymes:**		
PFD0830w *	bifunctional dihydrofolate reductase-thymidylate synthase (DHFR-TS)	3
PF13_0262	lysine-tRNA ligase, putative	409
PFI1310w	NAD synthase, putative	409
PFI0380c *	peptidyl deformylase (PDF)	2
PFA0480w	phenylalanyl-tRNA synthetase, putative	180
PFD0305c	vacuolar ATP synthase subunit b	112
**Transporter/Channels:**		
PF14_0244	ABC transporter, (EPP family), putative	637
PFE1150w	multidrug resistance protein (MDR1)	571
PF08_0113	vacuolar proton translocating ATPase subunit A, putative	876
**Other Proteins:**		
PFI0180w *	alpha tubulin 1	306
PFD1050w	alpha tubulin 2	857
PF10_0084 *	tubulin beta chain	74

a Identifiers and protein names taken from PlasmoDB [74]. Targets with known ligands according to TDR Targets database are marked with an asterisk (*) [27]. Essential kinases according to Solyakov *et al.* are shown in *italics* [18].

Note that the important role of both kinases and proteases was already suggested by Gamo *et al.* in their analysis of the TCAMS screening hits [8]. Among their 51 putative targets, they reported thirty kinases (59%) and ten proteases (20%). Keeping in mind that the set of phenotypically active compounds used in this work is mostly made of TCAMS compounds, it is remarkable to observe the correspondence of these percentages with those reported above based on our predictions. Yet, Gamo *et al.* did not provide any prioritization of their targets. In the present study, we recovered seven out of the ten proteases, all of them included in the high priority subset, and 16 out of thirty kinases, 13 of them as high priority targets.

More recently, Solyakov *et al.* reported a list of 36 *P. falciparum* kinases considered likely to be essential for intraerythrocytic asexual proliferation based on gene knock-out experiments [18]. A ligand-based target model was available for twenty of them. Remarkably, 18 are actually included in the predicted target space, with 13 in the high priority subset (shown in italics in Table 1) and an additional one being present in the set of high affinity targets.

### Target space of known antimalarial drugs

For comparison, a set of ten antimalarial drugs currently in clinical use and representative of all chemotypes shown in Figure 1 was also profiled *in silico*. Results are included in Table S1. From the group of hemozoin inhibitors chloroquine, mefloquine and quinine were considered. They are acting through the inhibition of hemozoin crystallization, a crucial step for detoxification of heme produced during haemoglobin degradation [2]. The assumed interaction partner of those drugs, the toxic heme species, is not present among the used target models and therefore cannot be predicted by the applied approach. Notably, the only target consistently assigned to all three drugs based on known interaction data is the multidrug resistance protein MDR1 (PFE1150w), which is associated with resistance issues of all of them [19], [20]. Beyond the interactions with MDR1, only quinine was predicted to address an additional target, namely the ABC sub-family G member 2 transporter (PF14_0244). The biological role of this transporter is not yet fully understood, but it could be speculated that it is involved in quinine resistance as well. Both MDR1 and the ABC transporter are contained in the set of high priority targets (Table 1).

The antifolates pyrimethamine, proguanil, and sulfadoxine were all assigned to their experimentally known target bifunctional dihydrofolate reductase-thymidylate synthase DHFR-TS (PFD0830w) [2]. In addition, sulfadoxine was assigned to dihydropteroate synthetase DHPS (PF08_0095), which is the primary target of this compound [21]. While DHFR-TS is one of the 39 high priority targets, DHPS was not predicted for any active compound. Apart from these experimentally known interactions, two additional new interactions were predicted. One is the interaction between pyrimethamine and MDR1. This is a potentially interesting prediction considering that neither the mechanism of action of this drug nor resistance has been related to this transporter so far. The other one is the interaction between sulfadoxine and acyl-CoA synthetase (PFF0945c), a protein included in the set of high affinity targets.

Atovaquone was used as a representative of the class of naphtoquinones. It is assumed to interact with the essential pyrimidine biosynthesis pathway of *P. falciparum* through the inhibition of cytochrome b (mal_mito_3). Furthermore, it is also known to bind dihydroorotate dehydrogenase (PFF0160c) involved in the same pathway [22], [23]. While both targets are contained in the predicted target space of 147 proteins, the latter one is the only target assigned to atovaquone by the *in silico* approach used here.

The antibiotics tetracycline and doxycycline inhibit protein biosynthesis via the inhibition of aminoacyl-tRNA binding to the ribosome [24]. Accordingly, these drugs were assigned to a set of five ribosomal proteins (PF14_0627, MAL7P1.93, PF11_0386, MAL13P1.92, PFE1005w), none of them part of the 147 targets predicted for the phenotypically active hits. Among the five predictions there is the putative apicoplast ribosomal protein S14p/S29e (PF11_0386). This is in line with the finding that such antibiotics specifically target the apicoplast of *P. falciparum* [25]. No additional targets were predicted for these compounds.

Finally, artemether was profiled as a representative of the artemisinin derivatives. The exact mode of action of this class of drugs is not yet known, even though multiple targets have been hypothesised [26]. In our hands, artemether is predicted to interact with MDR1 (PFE1150w), a reasonable prediction considering the fact that this transporter is associated with artemisinin resistance [19].

### Retrospective validation of the predicted antimalarial target space

An exhaustive prospective experimental validation of all predictions obtained by the presented computational approach is outside the scope of this study. Consequently, validation of our predictions at this stage is mostly done only retrospectively. A first immediate option is to examine the amount of experimentally known interactions between any of the orthologous model proteins corresponding to the 147 predicted *P. falciparum* targets and the 1,908 phenotypically active compounds predicted to have affinity to any of them. A total of 673 interactions were found to be contained already in the public repositories used for model generation. Among them, 411 interactions had an affinity value equal to or more potent than 10 µM, which were then compared with our predicted values obtained without considering them during model generation. Out of those 411 interactions, 223 (54.3%) were correctly predicted with an affinity value below 10 µM. For the remaining 188 interactions, 179 were in fact found outside the applicability domain. This result provides strong confidence in the predictions made.

An additional retrospective validation from a target side perspective is to select all *P. falciparum* proteins for which any known ligand exists based on the manual curation available at the TDR Targets database [27]. This yields a list of 85 targets, which can be regarded as the currently known part of the parasite's genome covered by small molecules. Of those, ligand-based models are available for 57 orthologous targets in our computational approach and could hence be part of our predictions. Remarkably, 51 of them (90%) are part of our predicted druggable *P. falciparum* genome of 226 targets, indicating that the chemical libraries screened by the St Jude, GSK, and Novartis groups cover potentially most of this druggable genome. Even more revealing is the fact that 36 of those targets (63%) are actually included in the target space of 147 *P. falciparum* proteins predicted to be addressed by phenotypically active compounds. It is worth stressing here again that ligand-based models are available for orthologous targets of 574 *P. falciparum* genes in the computational method used. The probability for a random target space of 147 proteins uniformly drawn from those 574 targets to contain at least 36 known druggable targets gives a p-value of 2.54×10^−10^. In fact, 15 out of the 36 recovered druggable proteins are contained in the set of 39 high priority targets, with an associated *p*-value of 6.07×10^−7^. These 15 targets are marked with an asterisk in Table 1. This means that our predicted *P. falciparum* target space shows a significant accumulation of known druggable targets, which provides further validation for our approach and its relevance for malaria drug discovery.

A final retrospective validation was performed against the current contents of the TDR Targets database that allows for prioritizing *P. falciparum* targets based on different features [27]. The query features were selected analogous to the example given by Agüero *et al.* in their supplementary information [27]. To establish a ranking of target relevance, they emphasize properties like assayability, essentiality, druggability, suitability for structure-based drug design and their phylogenetic distribution, among others (see Materials and Methods for details). A total of 5,349 *P. falciparum* targets were returned and prioritized according to their final cumulative weight. The TDR ranks of the 39 high priority targets are shown in Table 1. A total of 4 and 15 of our 39 high priority targets are ranked within the top-10 and top-100 targets by TDR, respectively. Conversely, among the top-39 targets ranked by TDR (which in fact contained 47 targets, several of which having the same rank), 9 were found in the set of high priority targets, 3 in the set of high affinity targets, 13 in the predicted antimalarial target space of 147 targets, and 11 in the predicted druggable genome of 226 targets. Only 11 targets (23%) were not present in any of our predicted or prioritized target sets. Altogether, this provides further evidence that the prediction and prioritization strategies used in this work lead to a malaria-relevant target space that complements nicely the target information currently available in TDR.

## Discussion

### Ligand-target malaria network

**Figure 4:**
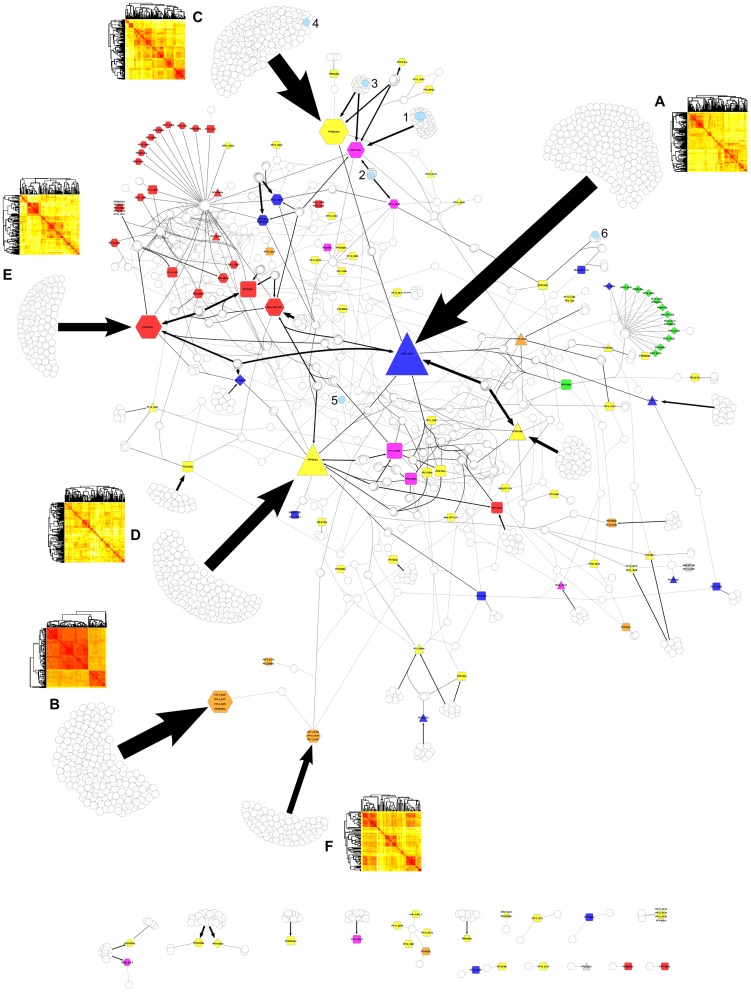
Drug-target network of 1,908 active compounds predicted for 147 *P. falciparum* proteins. Node colors encode target families (red: kinase; orange: protease; yellow: other enzyme; green: ribosomal protein; magenta: transporter/channel; blue: other protein; grey: unknown function), node shapes encode prioritization (hexagon: high priority; triangle: high affinity target; diamond: enriched target; square: other predictions). Targets from the same orthologous group are merged into one common node. Compounds are shown as white circles and grouped according to their target profiles. Edge width corresponds to the number of compounds in the respective group. Clinically used drugs are highlighted in light blue. The numbering corresponds to chloroquine, mefloquine, and artemether (1), quinine (2), pyrimethamine (3), proguanil (4), sulfadoxin (5), and atovaquone (6). Capital letters are used to identify the target hubs of Hsp90 (A), plasmepsin I, II, IV, and VI (B), bifunctional dihydrofolate reductase-thymidylate synthase (C), acyl-CoA synthetase (D), serine/threonine protein kinase ARK2 (E), and falcipain 2a, 2b, and 3 (F).

The complete ligand-target network connecting 1,908 phenotypically active compounds (white circles) with all 147 predicted plasmodial targets is presented in Figure 4. A fully scalable image is available as Supporting Information (Figure S1), allowing to zoom into individual targets to read their corresponding identifiers. The target nodes encode both the protein family (node color) and the priority class of the target (node shape). Targets belonging to the same OrthoMCL group share the same ligands and are therefore merged into a common node. The network contains also those clinically used drugs that were assigned to at least one of the 147 targets associated with phenotypically active hits, namely, chloroquine, mefloquine, artemether, quinine, pyrimethamine, proguanil, sulfadoxin, and atovaquone highlighted as light blue circles.

Up to 68% of all compounds displayed in the network are linked to a single target or target group. In fact, there are only six targets that can be recognized as strong network hubs, labeled with letters A to F in Figure 4: (A) Hsp90 (PF07_0029, linked to 204 compounds), (B) the plasmepsin group containing plasmepsin I, II, IV and VI (PF14_0076, PF14_0077, PF14_0075, PFC0495w, linked to 191 compounds), (C) bifunctional dihydrofolate reductase-thymidylate synthase (PFD0830w, linked to 189 compounds), (D) acyl-CoA synthetase (PFF0945c, linked to 148 compounds), (E) serine/threonine protein kinase ARK2 (PFC0385c, linked to 102 compounds) and (F) the falcipain group including falcipain 2a, 2b and 3 (PF11_0165, PF11_0161, PF11_0162, linked to 73 compounds). All but Hsp90 and acyl-CoA synthetase are contained in the list of 39 high priority targets collected in Table 1, which is due to the fact that these two targets do not appear to be enriched with phenotypically active compounds. Nonetheless, they are in the set of 59 targets identified as having a significant bias towards higher affinity predictions for phenotypically active compounds compared to the inactive ones.

A heatmap inset has been added in Figure 4 next to each of the six network hubs described above. These heatmaps illustrate the structural similarity between all compounds linked to each of those six target groups using pairwise Tanimoto indices calculated from their MACCS key fingerprints with Open Babel [28]. As can be observed, perhaps with the exception of the plasmepsin group, compounds appear organized in multiple small clusters of similar structures (red squares along the diagonal) that share only low to medium similarity with the remaining compounds of the same target group. Assuming that different chemical series may exhibit different binding modes with respect to the same target, this finding reflects the fact that affinity for those targets can be achieved with diverse chemotypes potentially having different target and ADME profiles, thus offering a wealth of opportunities to address drug resistance and safety issues.

### Multi-target malaria drug discovery

Most of the compounds are linked to one single target. It is worth stressing, however, that many compounds are involved in multi-target profiles, as it becomes apparent in Figure 4. Altogether they form a large interconnected network component containing 121 targets. In particular, kinases (shown as red nodes in Figure 4) form a tightly connected subnetwork. Indeed, in contrast to the traditional one drug – one target drug discovery paradigm, drug polypharmacology has gained a lot of attention in recent years [29]–[32]. For the particular case of malaria, small molecules acting with biologically relevant affinity on multiple *P. falciparum* targets represent an interesting strategy to retain therapeutic efficacy against the potential emergence of parasite resistance on individual targets, a concept already proposed for antimalarial protein kinase inhibitors [33], [34]. This is also supported by the observation that parasites show relatively slow resistance development against artemisinin, a drug that is believed to act on multiple targets [2]. Accordingly, phenotypically active compounds linked to multi-target profiles containing some of the identified high priority targets could be very attractive novel starting points for malaria drug discovery. The complete list of 272 predicted profiles of potential interest for malaria drug discovery is provided as Supporting Information (Table S2).

**Figure 5:**
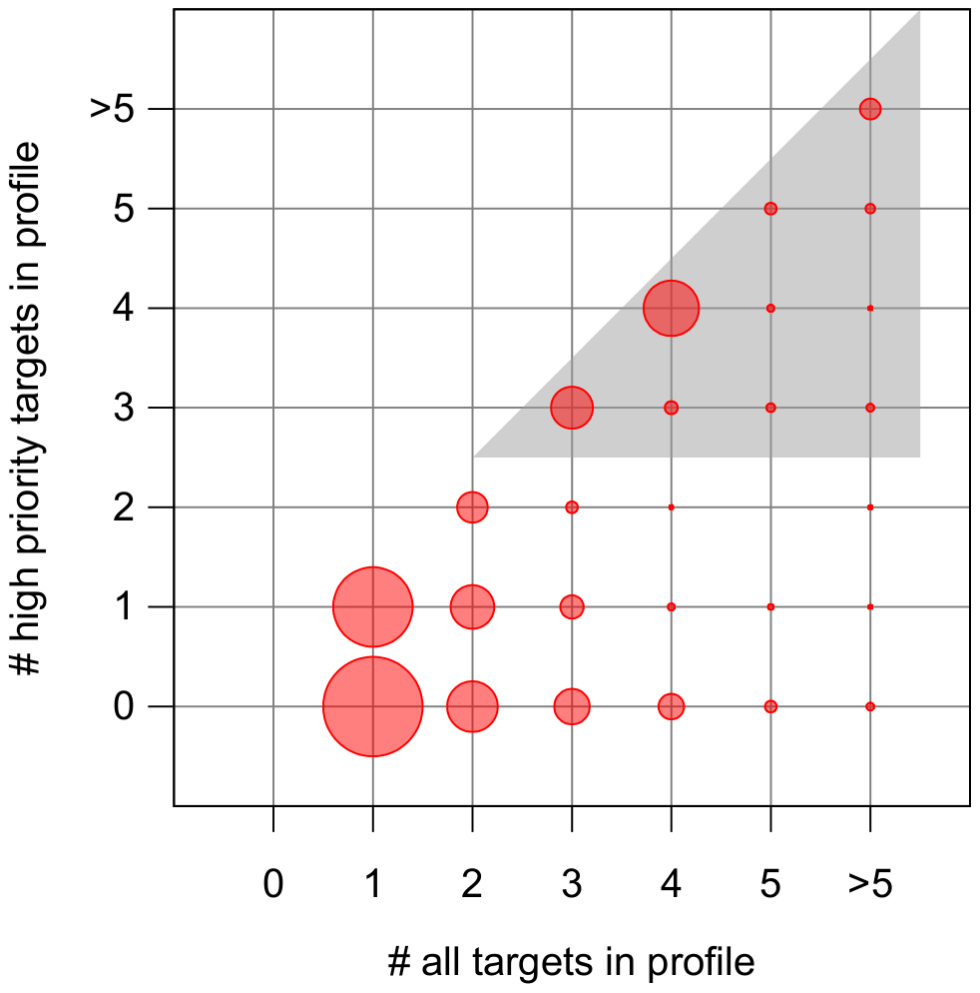
High priority targets versus total number of targets within a predicted profile. The size of a circle relates to the number of compounds that address a profile of the given characteristics.

Figure 5 shows the relationship between the target length of the 272 complete profiles predicted (x-axis) and the number of high priority targets included in them (y-axis). The size of the circles is proportional to the number of compounds assigned to a predicted profile of a given target length and number of high priority targets. The two large leftmost circles reflect the fact that the majority of the compounds are assigned to a single target. In general, it is observed that target profiles become enriched with high priority targets as the total number of targets in the profile increases. For example, among all compounds that have predicted affinities for three or more targets, 33% contain only up to two high priority targets, whereas more than twice as many (67%) contain three or more high priority targets in their profile (highlighted grey area in Figure 5). In contrast, it is also observed that about 48% of all compounds are not predicted to address any of the 39 high priority targets identified above (bottom row in Figure 5). However, among those compounds, 89% are predicted for at least one target that is either enriched or biased towards high affinity values for phenotypically active compounds.

In total, 64 unique profiles containing at least three targets, of which at least one is considered a high priority target, were identified (Table S2). The largest predicted target profile contains 31 targets, 25 of which are high priority targets. Among them, there are 20 kinases, three tubulins, a lysine-tRNA ligase and the multidrug resistance protein MDR1 (see Table 1). The compounds matching this profile are staurosporine and two close derivatives (GNF-Pf-1389, GNF-Pf-70 and GNF-Pf-3072). Staurosporine is a well-known kinase inhibitor that binds to many kinases with high affinity but little selectivity [35]. These three compounds are included in the upper-most circle. Several additional target profiles containing exclusively or predominantly kinases are also located in that profiling region.

The largest circle within the highlighted area in Figure 5 corresponds almost entirely to target profiles composed solely by the four high priority plasmepsin targets (Figure 4B). Since the four plasmepsins are contained in the same OrthoMCL group, a compound predicted for one of them is automatically assigned to all of them. Actually, this is a reasonable prediction since plasmepsin functionality is highly redundant within the *P. falciparum* food vacuole in which three of the four predicted plasmepsins are located. Due to this redundancy, it is unlikely that a selective plasmepsin inhibitor could stop parasite growth [36]. Thus, as compounds contained in this group are all known to be phenotypically active in a cell-based assay, there is a reasonable probability for them to be pan-plasmepsin inhibitors, consistent with the assignment to all members of the orthologous group. A similar argument can be made for the neighboring target profiles composed uniquely by three high priority targets. This circle contains compounds predicted to interact with either all three falcipain targets (Figure 4F) or all three tubulin targets (see Table 1).

**Figure 6:**
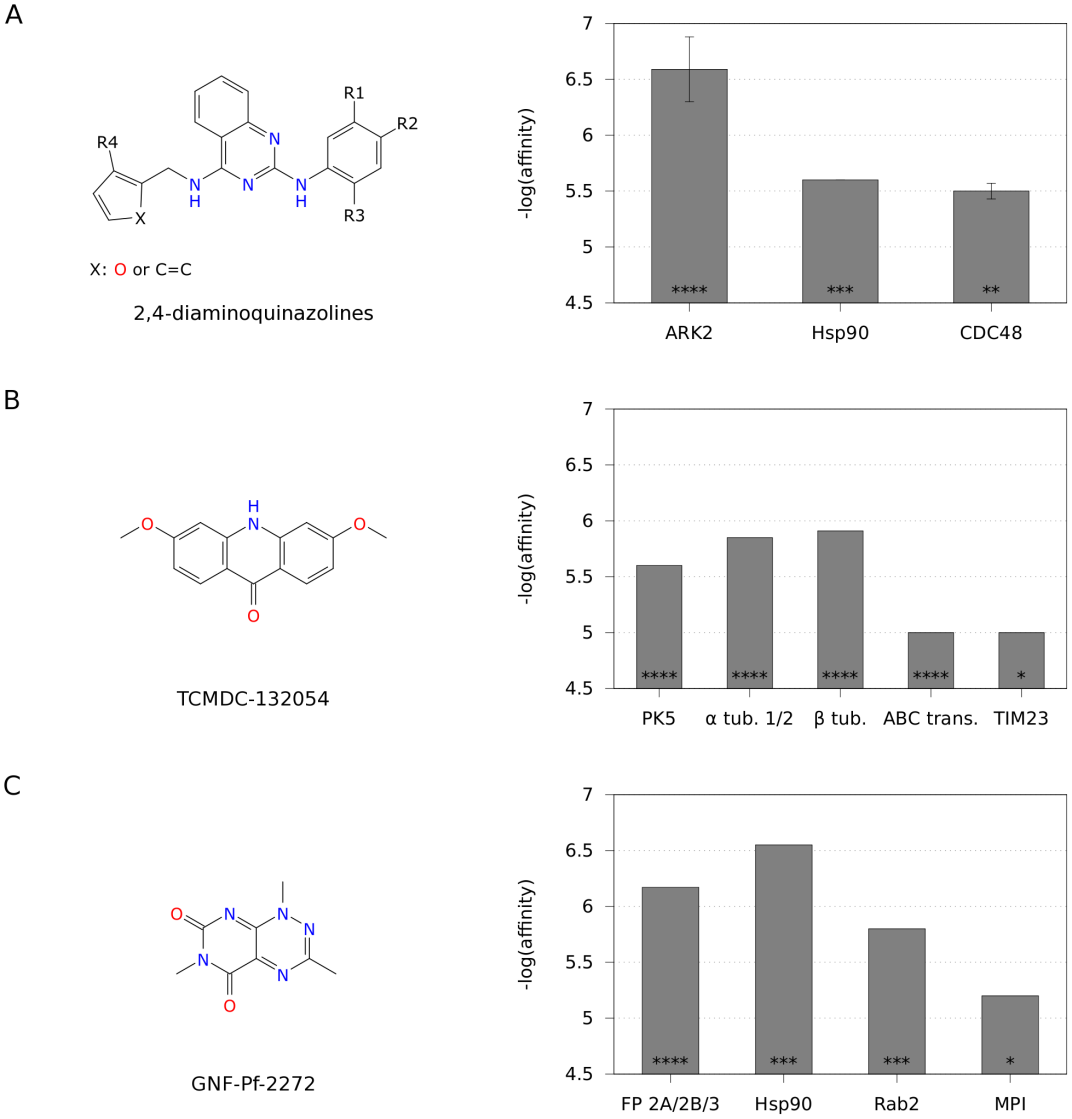
Selection of diverse multi-target profiles. The structures shown correspond to (A) the core scaffold of a set of 19 2,4-diaminoquinazolines, (B) TCMDC-132054, and (C) GNF-Pf-2272. The corresponding distributions of predicted affinities over multiple targets are provided on the right-hand side. For the set of 2,4-diaminoquinazolines (A), average predicted affinities are shown, with error bars giving the standard deviation over all compounds. Asterisks within the bars indicate the priority class of the respective target (‘****’ = high priority, ‘***’ = high affinity, ‘**’ = enriched, ‘*’ predicted target). Targets of the same orthologous group are joined to a single bar.

Beyond these family-directed kinase, protease and tubulin target profiles, a number of profiles composed of more diverse target combinations that can lead to novel opportunities for malaria drug discovery were also identified. Among them, a multi-target profile composed of three proteins predicted for a congeneric series of 19 2,4-diaminoquinazoline compounds is worth to be highlighted (Figure 6A). It comprises the serine/threonine protein kinase ARK2 (PFC0385c), heat shock protein 90 (Hsp90, PF07_0029) and a cell division cycle protein 48 homologue (CDC48, PFF0940c). While the exact biological function of ARK2 in the parasite is not yet known, this kinase is considered essential during the erytrothytic stage of the plasmodial life cycle [18]. In addition, a compound containing a similar diaminoquinazoline chemotype (CHEMBL552038) was found to inhibit the human orthologue Aurora kinase A with an experimental K_i_ value of 24 nM [37]. Kumar *et al.* showed that a known inhibitor of Hsp90, one of the most abundant chaperones in eukaryotes, is not only binding to the plasmodial protein but is also able to inhibit plasmodial growth, suggesting an essential role of Hsp90 in parasite development [38]. The role of the plasmodial CDC48 homologue is not yet discussed in the literature. However, orthologous proteins of several model organisms like *Saccharomyces cerevisiae*, have been shown to be essential [39]. The human orthologous valosin-containing protein (VCP) plays a crucial role in protein degradation through unfolding, segregating and translocating protein substrates [40], and is described to play an important role in guarding genome stability as it is part of an essential complex in ubiquitin-governed DNA-damage response [41]. In this respect, some compounds containing the exact diaminoquinazoline chemotype shown in Figure 6A (PubChem CIDs 46224519 and 46224526) have been already reported to inhibit VCP with IC_50_ values up to 0.8 µM [42]. Interestingly, four additional compounds containing a similar diaminoquinazoline chemotype are predicted for kinases GSK3 (PFC0525c, compounds TCMDC-135161 and TCMDC-134695), PK5 (MAL13P1.279, TCMDC-134695) and CLK1 (PF14_0431, GNF-Pf-2821 and GNF-Pf-4374). All of them are considered essential for erythrocytic parasite proliferation and diaminoquinazolines are known to inhibit at least the human GSK3β [18], [37]. This opens the possibility to add even more essential targets to the profile of the shown chemotype. The exact details of the complete multi-target profile associated with each individual compound within this series are provided in Supplementary Table S2.

Another interesting profile consisting of six targets (Figure 6B) includes protein kinase 5 (PK5, MAL13P1.279), the three tubulin targets alpha 1 (PFI0180w), alpha 2 (PFD1050w) and beta (PF10_0084), a putative ABC transporter (PF14_0244), all included in the high-priority set, and a subunit of the mitochondrial inner membrane translocase complex (PF13_0300). This profile was predicted for one compound (TCMDC-132054) but the target profiles of another six compounds containing the same acridinone chemotype were found to overlap partially, thus strengthening the signal for these predictions (see Table S2). Among the six targets, PK5 is considered to play a crucial role in the asexual proliferation cycle of intraerythrocytic parasite stages [18]. Also, tubulins are well-known essential antimalarial targets for parasite development, due to the fact that inhibition of microtubule formation affects cell division, motility and structural integrity of the parasite [43]. In fact, some of the closest similarity neighbors to TCMDC-132054 are 2-phenyl-4-quinolone derivatives, which are experimentally known to inhibit tubulin polymerization with submicromolar affinity [44]. The physiological role of the transporter PF14_0244 is not yet clear but the orthologous human ABCG2 transporter is involved in breast cancer drug resistance. Interestingly, one of the screened acrinidone derivatives (GNF-Pf-2549) was already shown to be able to inhibit the ABCG2 transporter with an IC_50_ value of 4.8 µM [45]. Finally, PF13_300 is a candidate for the TIM23 analogue in *P. falciparum*, a central part of the TIM23 pathway involved in protein import into the mitochondrial inner membrane and matrix in *S. cerevisiae* [46]. TCMDC-123947 is a close acridinone analogue of TCMDE-132054 known to inhibit *S. cerevisiae* TIM23 with an experimental IC_50_ value of 13.6 µM [47].

The last example of a multi-target profile of potential relevance to malaria (Figure 6C) contains the three cysteine proteases falcipain 2a (FP2A, PF11_0165), 2b (FP2B, PF11_0161) and 3 (FP3, PF11_0162), all belonging to the high-priority subset, two targets biased towards higher affinity values for active compounds, namely, the heat shock protein Hsp90 (PF07_0029) and the Rab GTPase Rab2 (PFL1500w), and mannose-6-phosphate isomerase (MPI, MAL8P1.156). Similarly to the previous case, this profile was predicted for one compound only, GNF-Pf-2272, but partial overlap is found in the predicted target profiles of three close pyrimidotriazinedione derivatives. It ought to be said that the affinities of GNF-Pf-2272 for human orthologues of two of the targets included in this profile are already known experimentally. These are Hsp90 and MPI, for which IC_50_ values are reported to be in the range of 400 nM and 6.1 µM, respectively [48], [49]. The role of Hsp90 in *P. falciparum* was already discussed above. Regarding MPI, its exact biological function in *P. falciparum* is not clear nor is it known to be essential. Yet, eukaryotic MPI in general is involved in several metabolic pathways. Among others, the product of MPI, mannose-6-phosphate, is acting as a signaling molecule for intracellular trafficking and MPI was shown to be essential for cell wall biosynthesis in several yeast species [50], [51]. As for the predicted affinities, the falcipains are known to play a crucial role in haemoglobin degradation, an essential process providing amino acids for protein biosynthesis during the parasites intraerythrocytic stages [52], [53]. Predictions were obtained for their human orthologues cathepsin L, cathepsin S and cathepsin K, with the close analogue GNF-Pf-67 being a known inhibitor of cathepsins S and K with IC_50_ values of 176 nM and 225 nM, respectively [54], [55]. Finally, Rab GTPases in eukaryotes are involved in the regulation of vesicular transport. With respect to Rab2 in particular, Quevillon *et al.* showed that this gene is transcribed in *P. falciparum* infected erythrocytes and they predicted a role in vacuole size regulation based on phylogenetic comparison to yeast [56].

For all targets involved in the presented multi-target profiles there exists experimental evidence that they might play central roles in the life cycle of the parasite. This evidence is either based on plasmodial proteins or orthologues from other species. In many cases there is also evidence available that the presented or closely related compounds are actually active on those targets. Therefore, the predicted profiles are reasonable and promising candidates for new starting points in the search for multi-target malaria drugs. However, it has to be mentioned that all those predictions were obtained from an orthologous projection to the plasmodial genome. Most of the original models were built from interaction data on human or other mammalian targets. Thus, the predicted compounds implicitly bear the risk for undesirable side effects due to the inhibition of host targets. This risk is even increasing with increasing profile sizes and, along with that, an increasing promiscuity of the compounds. For example, as mentioned above 2,4-diaminoquinazolines similar to those predicted for the profile in Figure 6A are already known to inhibit the human orthologues of ARK2 and CDC48 as well as additional human kinase targets. Moreover, compounds containing the 2,4-diaminoquinazoline scaffold are well known inhibitors of parasitic but also human DHFR [57]–[59].

The purpose of this study, however, was to deconvolute the molecular targets of non-optimized antimalarial HTS hits and to identify promising starting points for the development of novel multi-target malaria drugs. At this stage, this was done irrespective of any potential host interaction. Nevertheless, we strongly recommend keeping this inherent risk of adverse reactions in mind for any follow-up study. In particular when reaching the stage of lead optimization it remains to be seen whether these compounds can be developed into safer, more efficacious, drug candidates.

### Conclusions

Traditional malaria drug discovery has focused on the identification of small molecules that target individually some of the essential *P. falciparum* proteins identified to date. For example, chemotypes containing biguanides or aminopyrimidines are representative of an entire drug class targeting DHFR-TS (PFD0830w) and sulfonamides like sulfadoxine are designed to inhibit DHPS (PF08_0095). Indeed, with the exception of the artemisinin derivatives, all drugs are supposed to act very selectively on individual targets, which is fully supported by the above presented profiling results. Unfortunately, the long-term therapeutic usefulness of these chemical series has been severely hindered by the ability of the parasite to mutate and become resistant to those treatments. In this respect, artemisinin can be seen as the prototype of a multi-target malaria drug and the identification of a new generation of purposely designed multi-target drugs is emerging as an attractive strategy to overcome resistance by making more difficult for the parasite to evolve and survive to multiple mutations on essential proteins. However, the practicality of such a strategy is reduced to being able to prioritize, among the millions of possible combinations of *P. falciparum* proteins, those that could potentially lead to effective antimalarials. Beyond defining the *P. falciparum* target space likely to be most relevant to malaria drug discovery, the predictions derived in this work provide a first approximation to prioritizing the multi-target space addressed by phenotypically active antimalarials, thus paving the way towards more effective and robust malaria drugs.

## Materials and Methods

### Screening data sets

All processed data sets were downloaded from the ChEMBL – Neglected Tropical Disease archive [10]. The St. Jude set was divided into active and inactive compounds according to the 1,134 hits listed in the accompanying data sheet. Each data set was first filtered for duplicates. Then, the three sets of active hits were pooled and duplicates between the three sets were removed again. A set of 490 contradicting compounds, that is, compounds present in both the active and the inactive sets, were removed from the analyzed data sets. This happened in the case that a compound was found to be inactive by the St. Jude group but at the same time reported as a hit by the GSK or Novartis groups.

**Figure 7:**
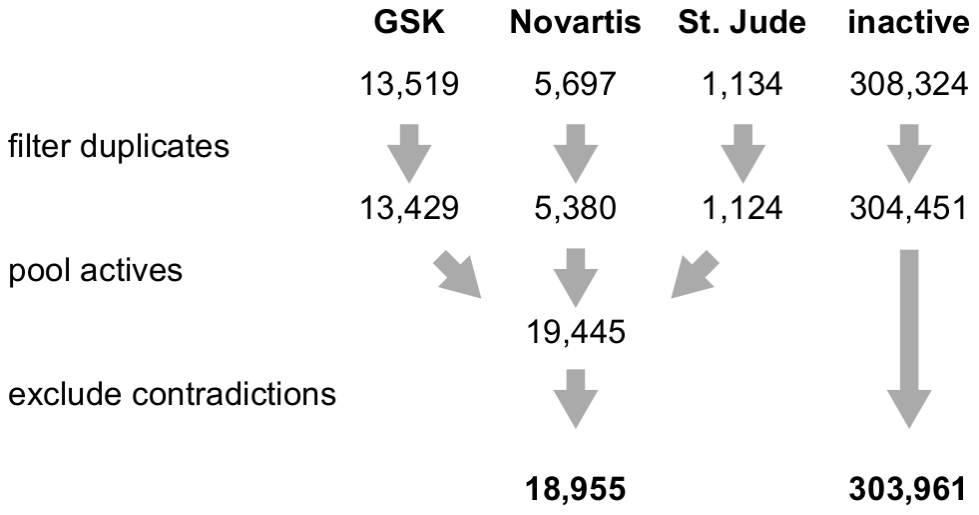
Data work-flow and library sizes during pre-processing of virtual target profiling.

Duplicates within and across data sets were filtered using Open Babel 2.3.0 [28]. Due to the fact that the topological descriptors applied in the subsequent target profiling (see below for details) do not distinguish stereo-isomers, such cases were treated as the same molecule during filtering. Contradictory assignments of the same molecule to both the active and the inactive set were detected in the same way. The whole pre-processing work-flow is shown in Figure 7.

### *In silico* target profiling

After pre-processing (Figure 7), both active and inactive molecules screened in phenotypic assays were processed with the target profiling approach implemented in the PredictFX software [16]. Given the two-dimensional structure of a molecule (smiles or sd/mol file), PredictFX returns the predicted affinities for those targets for which ligand information is available in public sources of pharmacological data [60]–[64]. Three ligand-based methods are implemented in the applied version of PredictFX that rely on descriptor-based similarities, fuzzy fragment-based mapping, and target cross-pharmacology.

Descriptor-based similarities are calculated using three types of two-dimensional descriptors, namely, PHRAG, FPD, and SHED [65], [66], each one of them characterizing chemical structures with a different degree of fuzziness and thus, complementing each other in terms of structural similarity and hopping abilities. Pharmacophoric fragments (PHRAG) are all possible fixed-length segments of five atom-features that can be extracted from the topology of a molecule. In contrast, feature-pair distributions (FPD) capture the overall spreading of pairs of atom-centered features at different predefined bond lengths. Finally, Shannon entropy descriptors (SHED) are derived from simplified FPD, in which, instead of using the actual feature-pair counts at each path length, the variability within all possible feature-pair distributions is quantified using the concept of Shannon entropy [66]. When using PHRAG and FPD, the similarity between two molecules corresponds to the overlapping fraction of their respective profiles [65], whereas with SHED, Euclidean distances are calculated instead [66]. All three descriptors were assessed on their ability to discriminate active from random compounds for all targets chemically represented in publicly available sources. As a result of this validation analysis, compounds below similarity values of 0.76 and 0.87 for PHRAG and FPD, respectively, and above a distance value of 0.52 for SHED were considered to be outside the applicability domain of these descriptors. For each of the 4,681 targets for which a PredictFX model was available, the ensemble of PHRAG, FPD, and SHED molecular descriptors of all known ligands represents a mathematical description of the target from a chemical perspective. On this basis, the affinity of a compound for a given target can be estimated by inverse distance weighting interpolation from the affinity landscape defined by all neighboring molecules according to the descriptors and similarity/distance metrics used [65], [66].

Fuzzy fragment-based mapping exploits the fact that, when a substantial chemical coverage is available for a given target, key interaction points can be revealed from the presence of specific chemical series with analogous scaffolds and multiple functionalities. Common trends within the same chemical series can be considered “primary” features, while the variable functionalities can be considered as “secondary” features. In this context, given a biological target, a simplest active subgraph (SAS) can be defined, which contains the minimum set of primary features required to achieve activity within a congeneric set of compounds. In order to generate a SAS model for a given biological target, all molecules with affinities below 1 µM are sorted according to their chemical complexity. Then, the simplest active molecule (SAM) is selected and molecules containing it to a certain degree of similarity are assigned to it. When all molecules have been processed, the next available SAM is selected and the process is iterated until all molecules are related to a SAM. The SAS identification protocol is not restricted to identical subgraphs. Instead, similar topologies can be identified, allowing a reasonable degree of scaffold hopping. Once the SAS model for a given biological target has been generated, it represents an alternative mathematical description of this target from a fuzzier ligand perspective and can be used for virtual screening purposes.

Finally, the target cross-pharmacology index between two targets A and B (XPI_A,B_) is defined as the fraction of compounds experimentally known to be active (pACT≥5.5) on target A and target B at the same time relative to all known ligands active on target A. If, for a given compound, an affinity to target A is predicted based on a SAS model, all cross-pharmacologically related targets B are identified for A. If no similarity-based or SAS-based affinity can be predicted for B, interaction affinities can be inferred for target B by using the corresponding cross-pharmacology index XPI_A,B_ as a weighting factor on the predicted affinity for target A. If several targets A* are related to B, then the inferred affinity for target B is the weighted average of all XPI_A*,B_ derived affinity values.

The method has been successfully validated retrospectively, on its ability to predict the entire experimental interaction matrix between 13 antipsychotic drugs and 34 protein targets [67] and to identify cancer-relevant targets from selective cytotoxic compounds in tumour cells [68], but also prospectively, on its capacity to identify the correct targets for all molecules contained in a biologically-orphan chemical library [69], to correctly anticipate the affinity profile of the drug cyclobenzaprine [70], to identify a confounding off-target of a widely used chemical probe [71], and to predict the target of novel inhibitors of amyloid β-induced neuronal apoptosis [72].

### Prioritization and *p*-value calculations

To identify targets with a statistically significant enrichment of active compounds among all their predictions, a permutation test was performed based on the null-hypothesis that predictions were uniformly distributed among all screened compounds. The respective *p*-Values were calculated according to the following formula:
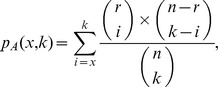
with *n* the total number of compounds (322,916), *r* the total number of actives (18,955), *k* the total number of predictions for a given target A and *x* the number of actives predicted for target A. Thus, *p*_A_(*x*,*k*) is the probability of getting at least *x* actives among the *k* predictions for target A, assuming that all possible combinations of actives and inactives among *k* are equally likely. If *p* is below a given confidence level the null-hypothesis can be rejected, meaning that it is reasonably unlikely to obtain the observed accumulation of active compounds by chance and the enrichment can be assumed to be significant. The same formula was used to calculate the probability of observing at least 36 and 15 out of 57 known druggable targets within a random target space of size 147 and 39, respectively.

To test for a location shift between the predicted affinity values for active and inactive compounds, a one-sided Wilcoxon rank-sum test was performed as implemented in the stats-package of R version 2.13.1 [73]. The test was based on the null-hypothesis that no shift between affinity values for the two samples of active and inactive compounds exists, and the alternative hypothesis that predicted affinities for active compounds are shifted towards higher values. In all statistical tests, a confidence level of 99.9% (*p*<10^−3^) was applied.

### TDR targets prioritization

In order to prioritize *P. falciparum* targets using TDR Targets database version 5, the following criteria were used (weights are given in brackets) analogous to Agüero *et al.*, supplementary figure S2: [27] Target species is *P. falciparum* (for all queries); target is an enzyme (100); molecular weight <100,000 (20); number of transmembrane segments = 0 (20); crystal structure of the target available (50); structure model available (30); ortholog present in all *Plasmodium* species (25); no ortholog present in *Homo sapiens* (25); any evidence of essentiality in any species available (40); druggability evidence index >0.6 (35); associated compounds known from manual curation (35); any form of validation data available (50); mapped publications available from PubMed (35). Targets ending up with the same final weight got the same rank. The search was performed on April 5th, 2013.

## Supporting Information

Figure S1 Fully scalable version of the ligand-target malaria network of 1,908 phenotypically active compounds (white circles) linked to 147 *P. falciparum* proteins presented in Figure 4. Capital letters are used to identify the target hubs of Hsp90 (A), plasmepsin I, II, IV, and VI (B), bifunctional dihydrofolate reductase-thymidylate synthase (C), acyl-CoA synthetase (D), serine/threonine protein kinase ARK2 (E), and falcipain 2a, 2b, and 3 (F).
(PDF)

Table S1 Complete predicted antimalarial target space of 226 *P. falciparum* proteins and their assigned compounds.
(XLSX)

Table S2 Complete list of target profiles predicted for phenotypically active antimalarials.
(XLSX)
