# Supplementary figures and images for: Prediction of the P. falciparum Target Space Relevant to Malaria Drug Discovery
Source: PLoS Comput Biol. 2013 Oct 17;9(10):e1003257. doi: 10.1371/journal.pcbi.1003257 (PMC3798273; doi:10.1371/journal.pcbi.1003257)

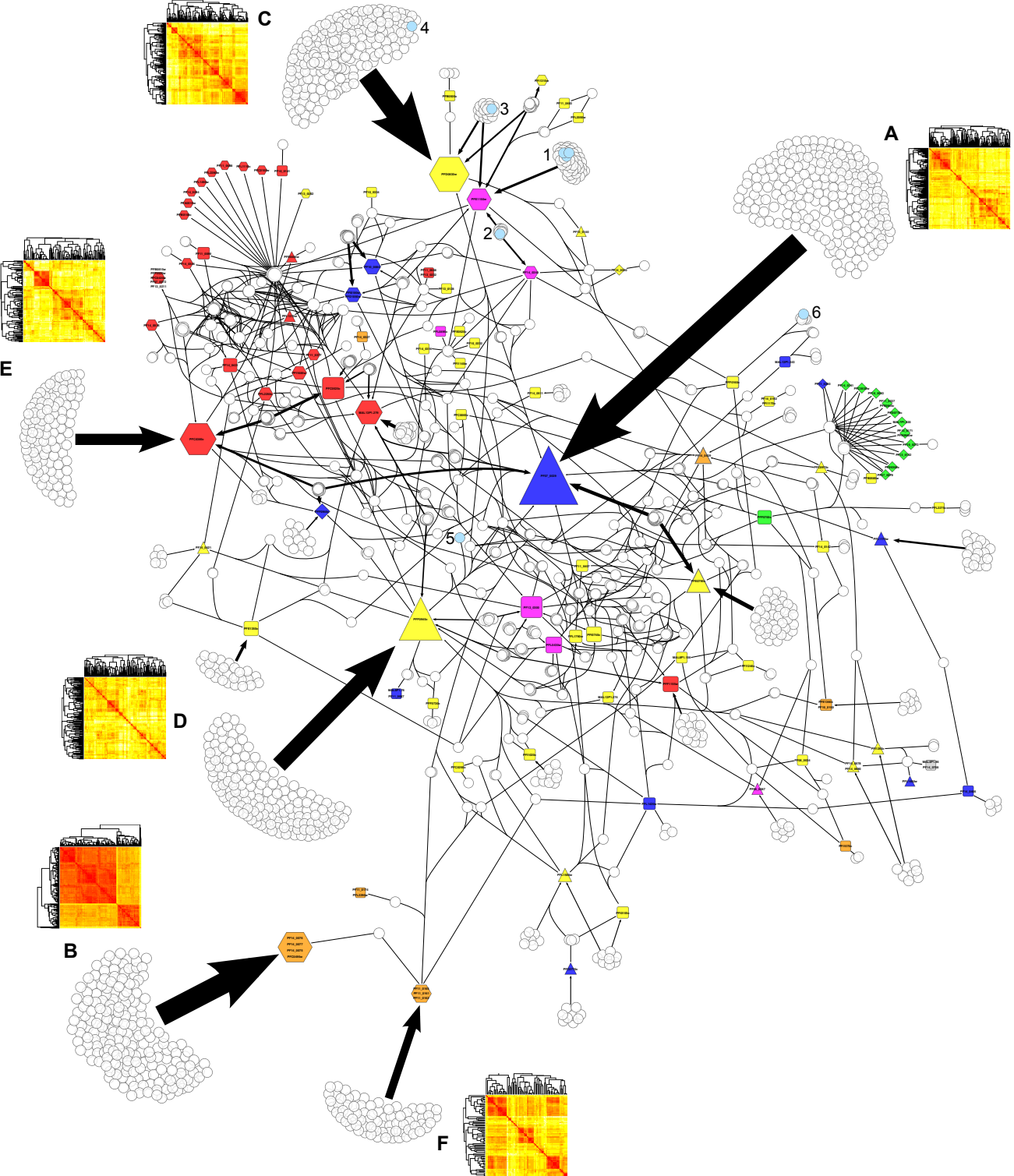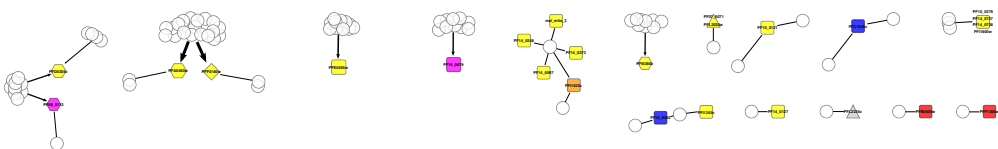

Supplement: Figure S1 — Fully scalable version of the ligand-target malaria network of 1,908 phenotypically active compounds (white circles) linked to 147 P. falciparum proteins presented in Figure 4. Capital letters are used to identify the target hubs of Hsp90 (A), plasmepsin I, II, IV, and VI (B), bifunctional dihydrofolate reductase-thymidylate synthase (C), acyl-CoA synthetase (D), serine/threonine protein kinase ARK2 (E), and falcipain 2a, 2b, and 3 (F). (PDF) [file pcbi.1003257.s001.pdf]
